# Supplementary material for: Causal Relationship between Aging and Anorexia Nervosa: A White-Matter-Microstructure-Mediated Mendelian Randomization Analysis
Source: Biomedicines. 2024 Aug 16;12(8):1874. doi: 10.3390/biomedicines12081874 (PMC11351342; doi:10.3390/biomedicines12081874)
Supplement: Supplementary file 1 [file biomedicines-12-01874-s001.zip › Supplementary Figure/Figure S4/Singe SNP plot of MR analysis for TL to ubm-b-1727.pdf]

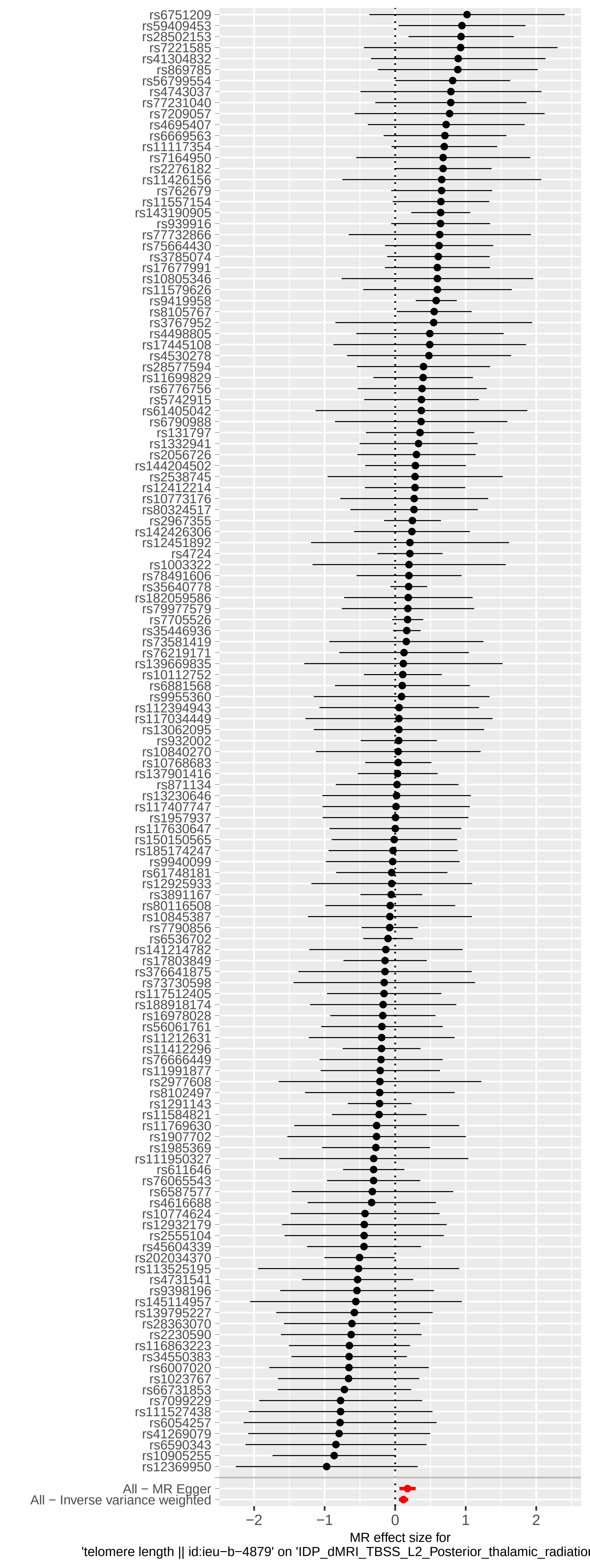
rs6751209
rs59409453
rs28502153
rs7221585
rs41304832
rs869785
rs56799554
rs4743037
rs77231040
rs7209057
rs4695407
rs6669563
rs11117354
rs7164950
rs2276182
rs11426156
rs762679
rs11557154
rs143190905
rs939916
rs77732866
rs75664430
rs3785074
rs17677991
rs10805346
rs11579626
rs9419958
rs8105767
rs3767952
rs4498805
rs17445108
rs4530278
rs28577594
rs11699829
rs6776756
rs5742915
rs61405042
rs6790988
rs131797
rs1332941
rs2056726
rs144204502
rs2538745
rs12412214
rs10773176
rs80324517
rs2967355
rs142426306
rs12451892
rs4724
rs1003322
rs78491606
rs35640778
rs182059586
rs79977579
rs7705526
rs35446936
rs73581419
rs76219171
rs139669835
rs10112752
rs6881568
rs9955360
rs112394943
rs117034449
rs13062095
rs932002
rs10840270
rs10768683
rs137901416
rs871134
rs13230646
rs117407747
rs1957937
rs117630647
rs150150565
rs185174247
rs9940099
rs61748181
rs12925933
rs3891167
rs80116508
rs10845387
rs7790856
rs6536702
rs141214782
rs17803849
rs376641875
rs73730598
rs117512405
rs188918174
rs16978028
rs56061761
rs11212631
rs11412296
rs76666449
rs11991877
rs2977608
rs8102497
rs1291143
rs11584821
rs11769630
rs1907702
rs1985369
rs111950327
rs611646
rs76065543
rs6587577
rs4616688
rs10774624
rs12932179
rs2555104
rs45604339
rs202034370
rs113525195
rs4731541
rs9398196
rs145114957
rs139795227
rs28363070
rs2230590
rs116863223
rs34550383
rs6007020
rs1023767
rs66731853
rs7099229
rs111527438
rs6054257
rs41269079
rs6590343
rs10905255
rs12369950
All − MR Egger
All − Inverse variance weighted
−2
−1
0
1
2
MR effect size for
'telomere length || id:ieu−b−4879' on 'IDP_dMRI_TBSS_L2_Posterior_thalamic_radiatio
